# Monitoring targets and indicators for the prevention and control of non-communicable diseases in Korea

**DOI:** 10.4178/epih/e2015023

**Published:** 2015-05-04

**Authors:** Soon Young Lee

**Affiliations:** Department of Preventive Medicine and Public Health, Ajou University School of Medicine, Suwon, Korea

**Keywords:** Chronic disease, Epidemiological monitoring, Health behavior, Policy

## Abstract

In order to respond to the increasing burden of non-communicable diseases (NCDs) worldwide, the World Health Organization developed the global action plan (GAP), which included nine targets and 25 indicators to monitor the targets. Owing to space constraints, the article reviewed the status of 17 indicators for seven targets out of nine targets in the GAP in Korea. Most of these 17 indicators required additional analysis with existing national data to evaluate the status in Korea. Based on the result from evaluating indicators, the current NCD policy strategy and resources in Korea seemed unlikely to reach the GAP goals, unless innovative policy changes was planned to reduce NCD risk factors particularly focusing on smoking, excessive drinking, and insufficient physical activity.

## INTRODUCTION

The World Health Organization (WHO) developed the global action plan (GAP) to reduce the burden of non-communicable diseases (NCDs) accounted for 65% of global deaths annually. The GAP suggested 25 indicators to monitor progress and to evaluate the nine targets set by GAP to reduce the global NCDs burden to be achieve by 2020 [1].

The monitoring would provide the assessment of NCDs trends between nations over time, which could lay a foundation for policy development and execution, and help increase political responsibility. In addition, the GAP proposed to develop additional indicators to monitor strategy development for NCDs prevention and control within each national and regional context. This included institutional reinforcement to increase the efficiency of data collection to generate indicators and improve the quality and scope of data [2]. The 25 GAP indicators include mortality and morbidity (indicators 1 and 2), NCDs risk factors (indicators 3-17), and national systems response (indicators 18-25). The national system response indicators were mainly patient treatments, drugs, and vaccines. This review focused on sources of data in Korea for the 17 indicators (indicators 1-17) related to NCDs mortality, morbidity and risk factors and highlighted the trend of each indicator.

## DATA SOURCE FOR MONITORING GLOBAL ACTION PLAN INDICATORS

Korean sources of data for the 17 indicators to monitor NCDs mortality, morbidity and status of risk factors were shown in Table 1. The major sources of data were the statistics from the Causes of Death Statistics, the National Cancer Registry, the Korea National Health and Nutrition Examination Survey (KNHANES), and the Korea Youth Risk Behaviors Web-Based Survey (KYRBS).

## GLOBAL ACTION PLAN INDICATOR LEVELS AND TRENDS

### Premature mortality due to non-communicable diseases

- Indicator 1. Unconditional probability of dying between 30 and 70 year-olds from cardiovascular diseases, cancer, diabetes or chronic respiratory diseases

- Indicator 2. Cancer incidence by type per 100,000 people

Indicators of premature mortality due to NCDs could be viewed as age-standardized mortality of individuals 30 to 69 years of age in causes of death statistics from the National Statistical Office of Korea. As the GAP included individuals up to 70 years of age, reanalysis is required. Nevertheless, premature death due to NCDs has been steadily decreasing in the past decade (Appendices 1-4). According to age-standardized cancer incidence by type from National Cancer Registry during the past 10 years, incidences of the six major cancer (gastric, lung, liver, colorectal, breast, and cervical cancer) have gradually increased, whereas the total cancer incidence has increased relatively faster. In particular, incidences of colorectal and breast cancer have increased more rapidly (Appendices 5 and 6).

### High-risk alcohol intake

- Indicator 3. Total alcohol per capita (15+ year olds) consumption

- Indicator 4. Age-standardized prevalence of heavy episodic drinking among adolescents and adults

- Indicator 5. Alcohol-related morbidity and mortality among adolescents and adults

Drinking-related indicators could be evaluated by data from the KNHANES, the Korea Alcohol Liquor Industry Association, and the Road Traffic Authority of Korea. There has been no major change in per-capita alcohol consumption over the age of 15 since 2007 (Appendix 7). The prevalence of high-risk drinking and the prevalence of alcohol abuse have been steady at about 14% since 2009 (Appendix 8) and 7% since 2008 (Appendix 9), respectively. However, the proportion of deaths due to driving under the influence of alcohol from the total traffic accidents has continuously increased, even though deaths due to traffic accidents have decreased steadily (Appendix 10). Mortality by mental or behavioral disorders caused by alcohol drinking has continuously decreased since 2000 while mortality by alcoholic liver diseases has rapidly increased until early 2000 and has been maintained at 7.5 per 100,000 persons for the last 10 years since 2004 (Appendix 11).

### Insufficient physical activity

- Indicator 6. Prevalence of insufficiently physically active adolescents

- Indicator 7. Age-standardized prevalence of insufficiently physically active persons aged 18+ years

Physical activity indicators could be evaluated using data from the KNHANES and KYRBS. Since age groups were differently categorized, reanalysis based on the definition of indicators for insufficient physical activities might be required. Physical activities in youths have gradually increased during the past five years, while physical activities among adults have decreased for the last 5 years (Appendices 12 and 13).

### Sodium intake

- Indicator 8. Age-standardized mean population intake of salt (sodium chloride) per day in grams in persons aged 18+ years

Sodium intake indicator could be calculated using data from the KNHANES, indicating an average intake of 6.5 g, which was 3 times higher than the 2 g target consumption of WHO. Despite a slight reduction in sodium intake compared to a decade ago, there has been no change during the past five years (Appendix 14).

### Smoking

- Indicator 9. Prevalence of current tobacco use among adolescents

- Indicator 10. Age-standardized prevalence of current tobacco use in persons aged 18+ years

Smoking indicators could be evaluated using data from the KNHANES and the KYRBS. Smoking rates among youths have been gradually decreased in the past five years, and smoking rates among adults have slightly decreased in the last decade (Appendices 15 and 16).

### Hypertension and diabetes

- Indicator 11. Age-standardized prevalence of raised blood pressure among persons aged 18+ years

- Indicator 12. Age-standardized prevalence of raised blood glucose/diabetes among persons aged 18+ years

Prevalence of hypertension and diabetes, which are biological risk factors, could be evaluated using the KNHANES data. The reanalysis is required to use 18 years as a starting age. The prevalence of hypertension has been increased during the past five years, while the previous trend of gradual increase in diabetes prevalence was decreased in 2012 (Appendices 17 and 18).

### Obesity

- Indicator 13. Prevalence of overweight and obesity in adolescents

- Indicator 14. Age-standardized prevalence of overweight and obesity in persons aged 18+ years

Obesity indicators could be evaluated by data from the KNHANES and the KYRBS. Although reanalysis of the past 10 years is required to use 18 years as a starting age for obesity in youths and adults and also the definition of obesity is different from that of WHO, obesity prevalence in youths and adults has been increased during the last decade (Appendices 19 and 20). As the KYRBS data was not based on actual measurements, data could be additionally supplemented using student health check-up data (Appendices 19 and 20).

### Additional indicators

- Indicator 15. Age-standardized mean proportion of total energy intake from saturated fatty acids in persons aged 18+ years

- Indicator 16. Age-standardized prevalence of persons (aged 18+ years) consuming less than five total servings (400 g) of fruit and vegetables per day

- Indicator 17. Age-standardized prevalence of raised total cholesterol among persons aged 18+ years and mean total cholesterol concentration

Although nutrient intake and dietary habit indicators were recorded in the KNHANES, saturated fatty acid intake was not officially reported until 2014. Recently, the Korea Centers for Disease Control and Prevention have developed methods to calculate intake of saturated fatty acids using the nutrients database by food types and these findings were expected to be presented in the near future. There have been no major changes in vegetables and fruit intake during the past five years. Recently, the KNHANES data has shown a continuous and rapid increase in the prevalence of high cholesterol (Appendices 21 and 22).

## EVALUATION OF THE 17 GLOBAL ACTION PLAN INDICATORS

Among the 17 GAP indicators described in the Table 2, five indicators were currently available based on the GAP definitions, but 11 indicators from the sources of data in Korea required the reanalysis based on age standards or GAP definitions. For the last indicator, intake rate of saturated fat has not been determined yet. It could be calculated by using the National Health and Nutrition Survey data in the near future.

## POSSIBILITIES OF ACHIEVING TARGETS ASSOCIATED WITH 17 GLOBAL ACTION PLAN INDICATORS

Of the nine GAP targets, seven targets corresponded to the 17 indicators described in this review. Under the assumption that the current national NCD policy in Korea would be maintained, the predicted chances to achieve the 2020 targets, based on the preceding trends of related indicators, were shown in Table 3. Although death indicators were most likely to meet the 2020 target, it would be a challenge for Korea to achieve many other risk factor-related targets. Therefore, innovative policy changes in Korea should be necessary to reduce NCD risk factors particularly focusing on smoking, excessive drinking, and insufficient physical activity.

## Figures and Tables

**Table 1.** Related statistics and data sources for global action plan (GAP) indicator monitoring in Korea

Related statistics for GAP indicators	Data sources
Indicator 1. Unconditional probability of dying between ages of 30 and 70 from cardiovascular diseases, cancer, diabetes or chronic respiratory diseases	Causes of Death Statistics (Appendices 1-4) [3]
Age-standardized premature mortality due to circulatory diseases (I00-I99) between 30-69 years of age	
Age-standardized premature mortality due to cerebrovascular diseases between 30-69 years of age	
Age-standardized premature mortality due to cardiovascular diseases between 30-69 years of age	
Age-standardized premature mortality due to neoplasm (C00-D48) between 30-69 years of age	
Age-standardized premature mortality due to diabetes (E10-E14) between 30-69 years of age	
Age-standardized premature mortality due to chronic lower respiratory diseases J40-J47) between 30-69 years of age	
Indicator 2. Cancer incidence by type of cancer per 100,000 people	National Cancer Registry (Appendices 5, 6) [4]
Total cancer incidence	
Six major cancer Incidence	
Cancer incidence by cancer types	
Indicator 3. Total (recorded and unrecorded) alcohol per capita (aged 15+ years old) consumption	Korea Alcohol Liquor Industry
Alcohol consumption per person aged 15 years or older	Association (Appendix 7) [5]
Indicator 4. Age-standardized prevalence of heavy episodic drinking among adolescents and adults	KNHANES (Appendix 8) [6]
Age-standardized high risk drinking prevalence among those 19 years or older	
Age-standardized annual drinking prevalence among those 19 years or older	
Age-standardized monthly drinking prevalence among those 19 years or older	
High risk drinking proportion from annual drinkers among those 19 years or older	
Indicator 5. Alcohol-related morbidity and mortality among adolescents and adults	
Alcohol abuse rate of lifelong drinkers	KNHANES (Appendix 9) [6]
Alcohol dependency rate of lifelong drinkers	
Proportion of deaths from accidents involving driving under the influence of alcohol	Traffic Accident Records (Appendix 10) [7]
Age-standardized mortality due to accidents	
Mortality by mental or behavioral disorder caused by alcohol drinking	Causes of Death Statistics (Appendices 10,11)
Mortality by alcoholic liver diseases	
Indicator 6. Prevalence of insufficiently physically active adolescents	KYRBS (Appendix 12) [8]
Prevalence of physical activities of at least five days per week and for 60 minutes and more per day among those 13-18 years of age	
Indicator 7. Age-standardized prevalence of insufficiently physically active persons aged 18+ years	KNHANES (Appendix 13) [6]
Age-standardized rate of more than moderate physical activities among those 19 years or older	
Age-standardized rate of more than moderate physical activities including walking among those 19 years or older	
Age-standardized rate of intense physical activities among those 19 years or older	
Age-standardized rate of moderate physical activities among those 19 years or older	
Age-standardized rate of walking among those 19 years or older	
Indicator 8. Age-standardized mean population intake of salt (sodium chloride) per day in grams in persons aged 18+ years	KNHANES (Appendix 14) [6]
Sodium intake among those age 9 or older	
Indicator 9. Prevalence of current tobacco use among adolescents	KYRBS (Appendix 15) [8]
Current smoking rates between ages of 13-18	
Indicator 10. Age-standardized prevalence of current tobacco use in persons aged 18+ years	KNHANES (Appendix 16) [6]
Age-standardized current smoking rates among those 19 years or older	
Indicator 11. Age-standardized prevalence of raised blood pressure among persons aged 18+ years	KNHANES (Appendix 17) [6]
Age-standardized hypertension prevalence among those 30 years or older	
Indicator 12. Age-standardized prevalence of raised blood glucose/diabetes among persons aged 18+ years	KNHANES (Appendix 18) [6]
Age-standardized diabetes prevalence among those 30 years or older	
Indicator 13. Prevalence of overweight and obesity in adolescents	KYRBS (Appendix 19) [8]
Obesity rate between 13-18 years of age	
Overweight rate between 13-18 years of age	
Indicator 14. Age-standardized prevalence of overweight and obesity in persons aged 18+ years	KNHANES (Appendix 20) [6]
Age-standardized obesity prevalence among those 19 years or older, based on body mass index	
Obesity prevalence among those 19 years or older, based on waist circumference	
Indicator 15. Age-standardized mean proportion of total energy intake from saturated fatty acids in persons aged 18+ years	KNHANES (not reported) [6]
Intake of saturated fats	
Indicator 16. Age-standardized prevalence of persons (aged 18+ years) consuming less than five total servings (400 g) of fruit and vegetables per day	KNHANES (Appendix 21) [6]
Age-standardized daily intake of vegetables (g) among those 1 year of age or older	
Age-standardized daily intake of fruits (g) among those 1 year of age or older	
Indicator 17. Age-standardized prevalence of raised total cholesterol among persons aged 18+ years and mean total cholesterol concentration	KNHKNANES (Appendix 22) [6]
Age-standardized high cholesterol prevalence among those 30 years or older	

Indicators 1-17 among 25 indicators in GAP are presented. KNHANES, Korea National Health and Nutrition Examination Survey; KYRBS, Korea Youth Risk Behaviors Web-Based Survey.

**Table 2.** Levels of generating global action plan (GAP) indicators in Korea^1^

Generating indicators	GAP Indicators^2^
Valid indicators have been generated and reported (5 indicators)	Indicator 2. Cancer incidence
	Indicator 3. Alcohol intake
	Indicator 9. Smoking rate of youths
	Indicator 13. Incidence of overweight and obesity in youths
	Indicator 14. Incidence of overweight and obesity in adults
Data sources and reported indicator levels exist, but minor revision is required (11 indicators)	Indicator 1. Mortality due to cardio-cerebrovascular diseases, cancer, diabetes, or chronic respiratory diseases
	Indicator 4. Incidence of excessive alcohol
	Indicator 5. Alcohol-related morbidity and mortality
	Indicator 6. Insufficient physical activity rates in youths
	Indicator 7. Insufficient physical activity rates
	Indicator 8. Daily sodium intake Indicator 10. Smoking rate
	Indicator 11. Hypertension prevalence
	Indicator 12. Diabetes incidence
	Indicator 16. Intake rates of fruits and vegetables
	Indicator 17. Excessive total blood cholesterol level
Data sources exist, but further analysis is therefore required (1 indicator)	Indicator 15. Intake rate of saturated fat

1 Indicators 1-17 of 25 total GAP indicators were shown.

2 GAP indicators were described in short.

**Table 3.** Possibilities to reach global action plan (GAP) targets in Korea [1]

GAP targets (-2020)	Possibility of achieving target
25% reduction of mortality by cardio-cere-brovascular diseases, cancer, diabetes, and chronic respiratory diseases	Possible
10% reduction of high risk alcohol intake	Little chance
10% reduction of insufficient physical activity rates	Little chance
30% reduction of mean salt intake	Little chance
30% reduction of current smoking rate in age 15 or older	Moderate chance
25% reduction of hypertension prevalence	Little chance
Suppression of the increasing diabetes and obesity trend	Little chance
